# Coping Intelligence: Efficient Life Stress Management

**DOI:** 10.3389/fpsyg.2017.00302

**Published:** 2017-03-03

**Authors:** Elena Libin

**Affiliations:** Independent Researcher, Chevy Chase, MDUSA

**Keywords:** life difficulties, coping Intelligence, coping IQ, efficient and inefficient coping, multidimensional positive coping model, temperament, life satisfaction

## Abstract

Coping Intelligence is defined as efficient individual ways of managing life stress. This paper presents a new assessment instrument named *Coping IQ (CIQ; Coping Intelligence Questionnaire)*. A measure is based on the Multidimensional Positive Coping Model, which includes three cross-cutting parameters that characterize coping strategy as efficient or inefficient, emotional, cognitive or behavioral, and active or passive. Results of the factor analysis verified a basic two-factor structure of the Coping Intelligence with the alternative solutions for efficient and inefficient coping strategies characterized via three basic modalities. The validity of the Coping IQ instrument showed an internal consistency ranging from 0.72 to 0.81. The unified methodology that underlies the new concept of Coping Intelligence, as well as *Coping IQ* assessment, is applicable for studying both clinical and general populations. *CIQ* parameters might serve as useful feedback while assessing changes in individual coping repertoire, for *CIQ* measures strategies that can be modified as a result of life experiences or educational training. Based on the study findings, Coping Intelligence is further defined by a broad repertoire of life skills required to solve successfully everyday stress and life adversities in order to achieve desired goals and maintain physical, mental, and social well-being.

## Introduction

‘Creating a science of human strength’ is a promising mission of psychology that focuses on ‘systematically building competence, not on correcting weakness’ (Seligman, 2000). This direction of research is based on a healthy, positive model of human behavior. The basic principals of positive psychology correspond strongly to the guidelines of differential psychology, whose primary goal is to explore the uniqueness of human individuality (Lamiell, 2003; Libin A., 2004). The concept of human competence is an ideal starting point for studying the complexity of human individuality and the fundamental issues of quality of life, including efficient and inefficient ways of coping with everyday difficulties that each of us faces on the way to happiness and personal achievements.

### Traditional Approach to Efficient and Inefficient Coping

There is a huge need among researchers, educators, academic professionals and practitioners, as well as among people of all ages, for knowledge on how to empower individual competence by mastering of efficient coping skills. However, existing studies on coping with life difficulties are very contradictory. Literature analysis of relevant concepts and related measures revealed two major trends in this research area: coping with stress (Lazarus and Folkman, 1984; Carver and Scheier, 1994) and applied problem solving (Heppner and Petersen, 1982; D’Zurilla and Nezu, 1990; Heppner et al., 2002, 2004). The most known in the first designated area of research is a cognitive theory of stress, developed by Lazarus and Folkman (1984), that interprets coping as either problem- or emotion-oriented. Problem-focused coping is directed toward managing and/or regulating a stressful situation. It occurs ‘if cognitive appraisal tells that something can be undertaken.’ Emotion-focused coping is directed toward regulation of emotional responses to a given stressor and occurs ‘when cognitive appraisal tells that nothing can be done’ in order to resolve a stressful situation (Folkman and Lazarus, 1985). This approach frames the development of The Ways of Coping (Lazarus and Folkman, 1984), which is a widely used instrument in health and clinical studies. Although this concept and the instrument proved to be very reliable in studying stress-evoked coping responses, a major limitation of this approach in the context of coping with everyday life difficulties is that emotional strategies are viewed as inefficient, whereas cognitive and behavioral strategies are always considered efficient.

A second trend in coping research emphasizes the importance of studying social aspects of problem solving competence through attitudes and underlying belief systems. An example is the social problem solving theory by D’Zurilla and Nezu (1990) who developed a Social Problem Solving Inventory (SPSI). This inventory consists of the problem solving skill scale (PSSS) and the problem orientation scale (POS) that includes cognitive, emotional, and behavioral subscales. Although the POS differs from the coping with stress theory (Lazarus and Folkman, 1984) in that it views cognitive and behavioral dimensions as separate categories, an emotional problem solving strategy still has the same negative connotation and is measured as an inefficient strategy.

A combined approach that merges the traditional applied problem solving concept and the stress-related coping theory resulted in Problem-Focused Style of Coping Scale (PF-SOC) developed by Heppner et al. (2004). The perceived effectiveness of a problem solving activity is viewed as the degree to which one’s actions facilitate or inhibit progress toward a resolution of the problem. The PF-SOC measures 18 strategies organized into three factors – reflective, reactive, and suppressive styles. *Reflective style* measures cognitive activities that promote problem solving, whereas *reactive style* emphasizes distorted cognitive and emotional activities. Denial and avoidance form *suppressive style*. Again, a cognitive strategy is analyzed as efficient or inefficient depending on the organization of the cognitive efforts, whereas emotional strategies along with behavioral ones, are defined as strictly inefficient.

### New Positive Coping Model: Efficient and Inefficient Management of Everyday Life Difficulties

The above described traditional approaches, while identifying cognitive, emotional, and behavioral aspects of coping, often confuse the modality of the strategy with its functionality and outcome. This conceptual drawback presents quite a few challenges to the measurement of efficient and inefficient strategies in coping research and psychological practice.

*First of all*, our review illustrates that the study of efficient and inefficient strategies has been limited in scope and in the choice of basic parameters. For instance, the inadequate conclusion that cognitive efforts are always efficient while emotional activities are always inefficient is based on a false assumption that basic parameters differentiating between efficient and inefficient strategies are associated with only one predominant modality. *Secondly*, existing models offer a very unclear depiction of the role of behavioral efforts. Behavioral strategies either form a separate category of inefficient coping (D’Zurilla and Nezu, 1990), or are combined with cognitive efficient strategies in one single class (Lazarus and Folkman, 1984; Folkman and Lazarus, 1988; Carver and Scheier, 1994). *Most importantly*, according to the existing approaches for problem solving and coping with stress, emotional strategies are viewed as contradictory to cognitive and behavioral ones. In the last two decades, numerous studies were conducted that proved the beneficial role emotions play in resolving life difficulties when they are used in manner appropriate to the task. Data suggest that particular characteristics of emotional experience as optimism, hope, and emotional intelligence positively influence the coping process (Seligman, 1991; Salovey and Sluyter, 1997; Snyder, 1998; Averill, 2000; Bar-On, 2000; Fredrickson, 2002). Salovey and Mayer (1990), defining the concept of Emotional Intelligence, stated that ‘emotion and intelligence are not mutually contradictory.’ As research shows, used accurately and adaptively, emotions help in reasoning, information processing, and problem solving by prioritizing thinking, shaping memory and facilitating creativity. Emotional strategies may be efficient if they are used adequately and inefficient if they employed inadequately for the process of resolving life difficulties. This findings support a new paradigm of understanding human intelligence that overcomes the limitations of ‘pure intelligence’ (Gardner, 1999) and its role in individual well-being. The contemporary view on individual competence considers emotional, cognitive, behavioral, and social abilities as integral parts of generalized intelligence (Goody, 1995; Salovey et al., 1999; Hedlund and Sternberg, 2000; Sternberg et al., 2003; Libin E., 2004). *In sum*, traditional perception of the incongruency between modalities (cognitive, emotional, or behavioral) of a particular strategy and its functionality or organizational efforts (efficient vs. inefficient) hinders the development of an integrated methodology for a generalized coping process and the design of an adequate assessment instrument.

A proposed concept of Coping Intelligence based on Multidimensional Positive Coping Model (Libin, 2003a) lays at the foundation of a new assessment instrument known as *Coping IQ (CIQ)*. This model strives to overcome traditional limitations in studying coping and problem solving by suggesting cross-cutting parameters for the unified classification of efficient and inefficient strategies. Coping Intelligence is defined by the quality, functionality, repertoire, and efficiency of cognitive, emotional, and behavioral strategies of varying intensity that people employ while dealing with difficult situations. Taking into account the new findings on generalized properties of human intelligence described in the previous section, the proposed model categorizes efficient and inefficient strategies based on *their functionality or the organization of coping efforts* (not on their modality). According to the Multidimensional Positive Coping Model each strategy is characterized by:

• ***The primary cross-cutting parameter****: organization of the efforts* (efficient or inefficient)
• ***The secondary cross-cutting parameter****: modality of manifestation* (emotional, cognitive or behavioral), and
• ***The cross-cutting parameter of the third level****: intensity of efforts* (active or passive)

Thus, *the organizational efforts* define a coping activity as efficient or inefficient, whereas *the modality* characterizes any given efficient or inefficient strategy as emotional, cognitive, or behavioral. In addition, each emotional, cognitive, or behavioral strategy can be evaluated as active or passive depending on the intensity of provided efforts. Hereby, a strategy is defined as a vector of emotional, cognitive, or behavioral efforts of varying intensity resulting either in an effective or ineffective outcome for dealing with life difficulties. Cognitive, emotional, and behavioral efforts that underlie efficient coping strategies *focus on* the resolution of the difficult situation. Accordingly, cognitive, emotional, or behavioral efforts underlying inefficient coping strategies *diverge from* the resolution of life difficulties.

Based on this model, a newly developed CIQ (Libin, 2002) differentiates between efficient or inefficient strategies as they relates to three basic modalities – cognitive, emotional, and behavioral, while including a measure of the intensity of human involvement with the situation, such as passive or active. The present article focuses on the first experimental phase of the new assessment tool development, whereas a theoretical foundation for the positive coping approach is described in details elsewhere (Libin, 2003a,b).

## Materials and Methods

Four consequential steps were performed in developing the *CIQ* assessment including (1) literature analysis and the development of a pool of items, (2) studying content validity of the new measure through the expert review panel, (3) exploring psychometric properties of the *CIQ* via Cronbach alphas, (4) and validation of the proposed measure via the analysis of individual differences in efficient and inefficient coping strategies with relation to age, gender, temperament, and subjective evaluation of meaningful life outcomes.

### Participants

The sample consisted of 114 participants with the mean age of 25.7 years including 28 (25%) males and 86 (75%) females. Participants were adolescents and young adults, recruited from public high school and colleges, and adults attending secondary education classes. The presented data is part of a larger cross-cultural study on coping with life difficulties currently being conducted in the U.S. Russia, and Ukraine.

#### Recruitment

Only participants who were enrolled in academic programs as part-time or full-time students were approached for informed consent. The recruiting process was conducted through a pre-screened list obtained from the Office of Academic Programs. All participants were assigned a number for the study, thereby maintaining their anonymity. Researchers involved with the project were trained and sensitized to the importance of confidentiality of the data.

### Measurement Instruments

The *CIQ* instrument was designed to measure cognitive, emotional, and behavioral responses to a difficult situation viewed as a meta-concept of problematic events that trigger coping efforts. *CIQ* is a self-report measure consisting of 72 items, which assesses three efficient and three inefficient scales differentiated by the cognitive, emotional, and behavioral modality of coping responses. The instructions ask a participant to indicate whether he or she employs a particular strategy while facing a difficult situation, using a 5-point Likert-type scale of frequency with ‘1 = never’ and ‘5 = always.’ Outcome variables included three measures of efficient and three measures of inefficient coping scales, two general indexes for efficient and inefficient strategies and four indexes for active and passive efficient and inefficient measures, as well as a combined quantitative measure named *coping intelligence quotation* calculated as a ratio of efficient strategies index divided by the inefficient strategies index. All indexes and scales were calculated as a mean of appropriate strategies. Each of six *CIQ* basic scales can be briefly described as follows:

• Efficient cognitive coping is characterized by cognitive activity *focused on* the resolution of the difficult situation, whereas inefficient cognitive coping characterizes cognitive activity *deviating from* the task at hand.
• Efficient emotional coping is comprised of emotional efforts *concentrated on* the problem’s solution, while inefficient emotional coping is associated with the emotional efforts *divergent from* resolving difficulties.
• Efficient behavioral coping consists of behavioral efforts *applied toward* resolving the difficulties. At the same time, inefficient behavioral coping characterizes behavioral activity *deviating from* problem-solving.

*Subjective Life Satisfaction Scale (SLS)* was developed and validated by the author in previous studies (Libin, 2003b). *SLS* measures subjective satisfaction with life goals, self and relationships with others on the 12 item Likert-type self-evaluation scale from ‘1 = completely dissatisfied’ to ‘5 = completely satisfied.’ Items refer to five separate, but interrelated aspects of one’s life – indexes of satisfaction with meaningful life outcomes (‘things that happened in my life,’ ‘projected goals,’ and ‘the way the life goes’), and indexes of satisfaction with socially oriented life areas including distant relationships (with superiors, colleagues, and peers) and close relationships (with friends, parents, and other family members) subscales. *SLS* also includes three single-items evaluating satisfaction with self, professional relationships, and relations with the opposite sex in general. *SLS* was tested on 60 people of both genders with the age mean of 27.4 years. Psychometric analysis showed a sufficient level of internal validity with the range of Cronbach alphas from 0.84 to 0.93 for scales and subscales combined as indexes.

*The Object-related and Communicative Temperament Inventory* (*STQ*; Rusalov, 1989) is based on the four-phase algorithm underlying Anokhin’s (1975) functional systems model. The *STQ* comprises 105 “agree-disagree” items organized in eight scales, measuring four basic temperamental parameters including ergonicity, plasticity, tempo and emotionality as they relate to social-oriented (communicative) and object-oriented areas of human activities. Four object-oriented scales measure ergonicity (Er), plasticity (P), tempo (T) and emotionality (Em) reflecting different aspects of mastering the object world. Social-oriented scales such as ergonicity (SEr), plasticity (SP), tempo (ST), and emotionality (SEm) measure, respectively, the level of social activity, the ease of switching from one social contact to another, the speed of social performance, and sensitivity in the communicative sphere. The *STQ* is shown to be a valid and reliable measure of temperament with Cronbach alphas ranging from 0.72 to 0.84 (Rusalov, 1989; Bishop and Hertenstein, 2004).

### Procedure

One hundred and twenty-eight participants were administered a set of three questionnaires over a 1-month period. A qualified researcher supervised the assessment performance. Each participant conducted self-evaluation individually. 14 participants were unable to compete the whole set due to the different reasons and were excluded from the data analysis. A total of 114 participants were included in the final analysis. Data were analyzed using SPSS 12.0.

### Analysis

The goal of the study was to address psychometric properties, as well as to analyze the structure of correlations between efficient and inefficient coping and other assessments, such as subjective life satisfaction and temperament. The content validity of the *CIQ* was studied through the experts’ panel. The reliability of the *CIQ* was assessed using an internal consistency measure based on Cronbach alphas. The construct validity of the developed coping measure was analyzed using a subjective life satisfaction scale and temperamental assessment. At the initial stage, descriptive univariate and bivariate statistics (e.g., frequency distributions, means, standard deviations, etc.) regarding participants’ background (gender and age) were examined. To further investigate the nature of efficient and inefficient coping strategies, we used correlations and an independent sample *t*-test, which clarified the reciprocal relationships between coping and background variables, and individual characteristics such as temperamental qualities and various aspects of subjective life satisfaction.

### Results

The study of content validity of the *CIQ* was conducted through the expert review panel, which included four experts familiar with the literature on coping. All experts were psychologists and academic professionals experienced at teaching high school, undergraduate and adult students. The panel reviewed all *CIQ* items prior to the testing. Necessary word changes were made so that the proposed items would be better understood by the participants. Then experts reviewed the list of items, rating relevance of the items to efficient or inefficient coping. The initial pool for the questionnaire included 180 items, which after initial reviewing with the group of four researchers was narrowed down to 108 items. During the next step an internal consistency of the *CIQ* was studied via data collected from 114 participants. As a result 36 more items were excluded, leaving 72 items with most significant loading organized in six scales with Cronbach alphas ranging from 0.72 to 0.81 (**Table 1**).

**Table 1:** *Coping IQ (CIQ)* scales reliability via Cronbach alphas.

CIQ Scales	CIQ item sample	Mean	*SD*	α
Inefficient cognitive	Get caught up in thinking about insignificant details	2.62	0.55	0.75
Inefficient emotional	Feel that I will never get over it	2.36	0.57	0.76
Inefficient behavioral	Do anything but the task at hand	2.10	0.51	0.72
Efficient cognitive	Break up the complex problem into simple manageable components	3.67	0.55	0.78
Efficient emotional	Use my desires and interests as direction where I want to go in solving the difficulty	3.61	0.59	0.81
Efficient behavioral	Work through the difficulties until the situation is completely resolved	3.63	0.52	0.80

The next step was to study a structure of the *CIQ* via factor analysis. We assumed that two basic dimensions, inefficient and efficient coping, would be associated with two different factors. This structure of the *CIQ* was confirmed by the principal component factor analysis with Varimax rotation of 72 items. The result revealed a basic two-factor structure with the alternative factor solutions for efficient and inefficient coping strategies. Each basic factor (efficient or inefficient) included all three modalities; that is, cognitive, emotional, and behavioral scales.

The relationship between efficient and inefficient coping strategies, measured by *CIQ*, and gender, age, and individual characteristics (such as temperament and life satisfaction) were studied on groups of 61 and 70 people, respectively. Additionally, gender differences were studied on the group of 48 people (24 male and 24 female) aged 14–17.

#### Gender and Age Differences in Efficient and Inefficient Coping

Analysis via independent sample using Levene’s test for equity of variances as a statistical measure (F) of the differences between the groups (*N* = 48) showed no statistically significant gender-related differences regarding the preference of efficient vs. inefficient strategies. Comparison by Levene’s test between two age groups of adolescents (15–16 year old, *N* = 31) and young adults (17–21 year old, *N* = 36), both balanced by gender, revealed significant differences in inefficient emotional and efficient cognitive coping scales. Additionally, comparisons revealed differences in the integrative coping intelligence quotation, as well as in the intensity of coping efforts measured through indexes of active and passive strategies. Only outcomes with an alpha level of less than 0.05 were considered for interpretation. Distribution of the analyzed variables was fairly symmetric and had no outliers (**Table 2**).

**Table 2:** Age differences in efficient and inefficient coping.

Scale	Group	Mean	*SD*	*F*	*p*
Inefficient emotional	*N* = 31	2.54	0.73	7.02	0.009
	*N* = 36	2.39	0.47		
Efficient cognitive	*N* = 31	3.47	0.67	5.04	0.028
	*N* = 36	3.71	0.45		
**Index**					
Inefficient active scales	*N* = 31	2.53	0.55	6.56	0.013
	*N* = 36	2.46	0.38		
Inefficient passive scales	*N* = 31	2.49	0.58	8.90	0.004
	*N* = 36	2.41	0.43		
Efficient active scales	*N* = 31	3.47	0.66	4.41	0.040
	*N* = 36	3.69	0.41		
Coping intelligence quotation	*N* = 31	1.44	0.43	8.71	0.004
	*N* = 36	1.53	0.25		

Correlation analysis of the coping, subjective life satisfaction and temperamental parameters, measured, respectively, via *CIQ*, *STQ*, and *SLS*, confirmed our initial hypothesis about the links between inefficient strategies, life dissatisfaction, and temperamental impulsivity and anxiety (**Table 3**).

**Table 3:** Correlations between coping strategies, subjective life satisfaction, and temperament.

Parameter	InCoSc	InEmSc	InBhSc	EfEmSc	EfBhSc	Inefficient coping	Efficient coping	Coping quotation
***SLS***								
S5		-0.37ˆ**	-0.28ˆ*			-0.33ˆ**		
S11		-0.36ˆ**				-0.30ˆ*		
IMLO		-0.39ˆ**	-0.26ˆ*			-0.30ˆ*		
ISR	-0.26ˆ*	-0.45ˆ**	-0.31ˆ**			-0.40ˆ**		
**STQ**								
SEr	-0.26ˆ*							
SP				0.32ˆ*	0.29ˆ*		0.31ˆ*	0.34ˆ*
T				-0.26ˆ*				-0.26ˆ*
ST	-0.27ˆ*		-0.29ˆ*			-0.30ˆ*		
Em	0.29ˆ*	0.27ˆ*	0.26ˆ*			0.31ˆ*		
SEm	0.38ˆ**	0.48ˆ**	0.36ˆ**			0.47ˆ**		-0.36ˆ**
ISAct	-0.43ˆ**	-0.30ˆ*	-0.32ˆ*			-0.40ˆ**		0.35ˆ**

*Coping Inventory (CIQ) scales and indexes (N = 70): InCoSc, Inefficient Cognitive Scale; InEmSc, Inefficient Emotional Scale; InBhSc, Inefficient Behavioral Scale; EfEmSc, Efficient Emotional Scale; EfBhSc, Efficient Behavioral Scale; InCoping, Inefficient Coping Index; EfCoping, Efficient Coping Index; Coping IQ, Coping Intelligence Quotation. Subjective Life Satisfaction Scale (SLS) (N = 70): S5, Satisfaction with Professional Relationships; S11, Satisfaction with the Opposite Gender Relationships; IMLO, Index of Satisfaction with Meaningful Life Outcomes; ISR, Index of Satisfaction with Social Relationships in General. Temperament Inventory (STQ) scales and indexes (*N* = 61): Er, Object-oriented Ergonicity Scale; P, Object-oriented Plasticity Scale; T, Object-oriented Tempo Scale; SEr, Social-oriented Ergonicity Scale; SP, Social-oriented Plasticity Scale; ST, Social-oriented Tempo Scale; ISAct, Index of Social-oriented Activity; IEm, Index of Object-oriented Emotionality. ^∗^p < 0.05, ^∗∗^p < 0.01.*

Results of the correlation analysis showed that a higher index of *inefficient coping* via *CIQ* was associated with the lower levels of meaningful life outcomes, including goals, major life events and future prospects, personal well-being, and social relationships. The largest number of significant correlations between *ineffective coping* and low scores on *STQ* was found for the parameters of temperamental emotionality (neuroticism) and tempo (impulsivity). Cognitive, emotional, and behavioral ineffective coping strategies were also associated with subjective dissatisfaction in various domains of life. The general index of inefficient coping, measured as a mean of all three scales, correlated negatively with major subjective satisfaction parameters. Statistically significant links were found between coping strategies and all temperamental parameters assessed via *Temperament Inventory (STQ), with the exception of* object-oriented ergonicity and plasticity. In sum, *inefficient coping was found to* correlate positively with temperamental emotionality and negatively with social tempo and social activity. *Efficient coping* correlated positively with both socio-oriented plasticity and temperamental activity, while negatively with neuroticism (**Table 3**).

A *T*-test was performed to clarify the structure of the relationships between different levels of coping intelligence quotation, temperament, and life satisfaction. The comparative analysis of groups (mean age 23 years) with high and low levels of Coping IQ by temperament and subjective life satisfaction revealed that individuals with *efficient coping* are characterized by a higher level of *social-oriented plasticity* (*t*_(39)_ = −3.05, *p* < 0.04) and *index of social-oriented activity* (*t*_(39)_ = −3.36, *p* < 0.02). Individuals with *inefficient coping* are distinguished by the higher level of *object-oriented tempo* (*t*_(39)_ = 2.14, *p* < 0.04).

Also, participants with high levels of *inefficient coping* are characterized by an increased level of *dissatisfaction with meaningful life outcomes* (*t*_(34)_ = −2.47, *p* < 0.02), *social relationships* in general (*t*_(34)_ = −2.56, *p* < 0.02), and *distant social relationships* in particular (*t*_(34)_ = −2.53, *p* < 0.02). Individuals with low Coping IQ are more dissatisfied with major aspects of life, including ‘things that happened in life’ (*t*_(34)_ = −1.08, *p* < 0.05), ‘projected goals’ (*t*_(34)_ = −2.22, *p* < 0.03), and ‘the way life goes’ (*t*_(34)_ = −1.99, *p* < 0.05). In socially oriented areas they are especially unhappy with their distant relationships, including those with superiors (*t*_(34)_ = −2.62, *p* < 0.01), colleagues and peers (*t*_(34)_ = −2.00, *p* < 0.02), with their parents (*t*_(34)_ = −2.48, *p* < 0.05), and with their relationships with the opposite gender (*t*_(34)_ = −2.06, *p* < 0.05).

The general conclusion is that a *low Coping IQ*, is associated with a predominance of ineffective strategies in individual repertoire, and is linked with high scores on such individual variables as temperamental impulsivity (tempo) and subjective dissatisfaction with personal achievements and with relationships with others. On the contrary, a *high coping intelligence quotation*, associated with a predominance in individual repertoire of effective strategies, is linked to socio-oriented temperamental flexibility (plasticity) and subjective satisfaction with both personal achievements and with social aspects of life.

## Discussion

### Coping Intelligence Concept

Both academic researchers and practitioners have noted that the absence of general principles for classification of efficient and inefficient strategies poses methodological and practical difficulties in their diagnostics and differentiation, thereby causing additional obstacles in the systematic study of this important phenomenon (Chang et al., 2004). The newly developed concept of Coping Intelligence suggests the use of cross-cutting parameters to facilitate the unified classification of efficient and inefficient coping. Results of the factor analysis verified a basic two-factor structure of Coping Intelligence with alternative solutions for efficient and inefficient strategies characterized via three basic modalities.

### Coping IQ Assessment

A theorized relationship between efficient and inefficient coping strategies, positioned in the continuum formed by three basic dimensions – cognitive, emotional, and behavioral– guided the development of the *CIQ* instrument that was designed to measure a variety of strategies in all three dimensions that individuals tend to use while facing life difficulties. While the primary cross-cutting parameter (*organization of the efforts)* differentiates between effective and ineffective strategies, the secondary cross-cutting parameter describes each efficient and inefficient strategy as cognitive, emotional, or behavioral according to *the manifest modality* of the efforts.

The final version of the *CIQ* instrument consists of 72 selected items to ensure high reliability for each of three effective and three ineffective scales. Outcome variables included six *CIQ* basic scales, two general indexes for efficient and inefficient strategies, and coping intelligence quotation calculated as a ratio of *efficient coping index* divided by the *inefficient coping index*. As a quantity indicator, *Coping IQ* shows whether efficient coping strategies prevail in the individual’s repertoire.

### Relationships between Efficient and Inefficient Coping, Temperament, and Subjective Life Satisfaction

Results showed that the young adults employ efficient strategies more often than the teenagers. Changes in coping related to age dynamics suggest that individual efficient coping repertoire arises initially as a result of the development of emotional and cognitive mental processes. Our findings also confirmed that not only emotional, but also cognitive and behavioral inefficient strategies are associated with low life satisfaction.

The association between inefficient coping strategies and *object-oriented temperamental impulsivity (high tempo)* corresponds with the data on increased problematic behaviors in persons with high levels of impulsivity (Horton and Oakland, 1997; Mcevoy and Welker, 2000). This allows us to make an assumption that higher psychomotor activity negatively influences coping outcomes. It is not the speed of object-oriented mental operations and motor acts performance (indicators of *high tempo* or *impulsivity*), but rather the accuracy with which mental and motor activity are performed (*adequate and timely channeled tempo)* along with the plasticity of social-oriented activity that contributes to the successful resolution of life difficulties. In the realm of social relationships, a broader repertoire of communicative programs, and flexibility in social relationships and in establishing social contacts (indicators of high *social plasticity)* are most likely to result in more efficient ways of dealing with other people which. This, in turn, could ease the complex process of handling life challenges.

The greater number of statistically significant correlations between temperamental (formal-dynamic) characteristics and inefficient strategies vs. efficient coping demonstrates close ties between formal-dynamic, biologically determined variables and inadequate ways of dealing with difficult situations. At the same time, *efficient* and *inefficient coping strategies* demonstrate reverse relations with both temperamental (formal-dynamic) and subjective life satisfaction (socio-psychological) characteristics. In comparison to efficient coping, inefficient strategies also revealed a much greater extent of negative association with subjective satisfaction parameters, thus illustrating the greater impact of inefficient coping on personal dissatisfaction with various aspects of life.

## Ethics Statement

This study was carried out in accordance with the recommendations of Institutional Review Board at the Institute of Psychology, Russian Academy of Education. All subjects gave written informed consent in accordance with the Declaration of Helsinki. The protocol was approved by the Institutional Review Board at the Institute of Psychology, Russian Academy of Education.

## Author Contributions

The author confirms being the sole contributor of this work and approved it for publication.

## Conflict of Interest Statement

The author declares that the research was conducted in the absence of any commercial or financial relationships that could be construed as a potential conflict of interest.
